# A systematic review, bibliometric analysis, and meta-analysis of breed adaptation and sustainable systems for enhanced beef cattle performance in Mozambique

**DOI:** 10.1007/s11250-026-05445-4

**Published:** 2026-11-04

**Authors:** Virgilio Juma Ali, Giulia Correa Sassi, Tadayuki Yanagi Junior, Alessandro Torres Campos, Arsenio Mario Caetano, Sergio Abilio De Azevedo

**Affiliations:** 1 Department of Engineering, Federal University of Lavras, Lavras, Minas Gerais 37000-200 Brazil; 2 Department of Forest Sciences, Federal University of Lavras, Lavras, Minas Gerais 37000-200 Brazil

**Keywords:** Indigenous cattle, Landim, Nguni, Climate resilience, Smallholder systems, Sub-Saharan Africa

## Abstract

Beef cattle production in Mozambique faces productivity constraints due to heat stress, limited adoption of sustainable practices, and use of non-adapted breeds. This study integrated systematic review (*n* = 57 publications), bibliometric analysis, and mixed-effects meta-analysis (*n* = 15 studies, 578 animals) to quantify the effects of breed adaptation and production system sustainability on daily weight gain in Mozambican cattle. Adapted breeds (Landim, Nguni, Angoni) showed significantly higher weight gain (+ 0.278 kg/day, *p* < 0.05) compared to non-adapted breeds, while sustainable management practices (shading, rotational grazing, improved feeding) added + 0.194 kg/day. The final predictive model explained 98.3% of observed variability (R²=0.983) with low prediction error (RMSE = 0.124 kg/day). Adapted breeds under sustainable systems achieved the highest predicted weight gain (0.78 kg/day), approaching commercial viability (0.8 kg/day), whereas non-adapted breeds under traditional systems showed the lowest performance (0.31 kg/day). Geographic distribution revealed research concentration in southern/central regions (82.5% of studies) and underrepresentation of northern Mozambique. These findings demonstrate synergistic benefits of aligning genetic adaptation with sustainable management, supporting evidence-based policies for resilient beef production in Mozambique and similar tropical systems.

## Registration and protocol

This systematic review was not registered. A protocol was not prepared for this review.

## Introduction

Beef cattle production plays a central role in Mozambique’s agricultural economy, contributing to rural livelihoods, national food security and the functionality of regional value chains (Rafael 2012, Macia and Filimone 2024). Despite this importance, the sector continues to face persistent limitations, including low productive efficiency, restricted access to improved genetics, climate-related stress and insufficient integration of sustainable management practices (Vernooij et al. 2016, Carvalheira et al. 1995, King et al. 2022). These challenges collectively reduce productivity and compromise the long-term resilience of production systems (Ngxumeshe et al. 2020).

Genetic adaptation is a fundamental determinant of performance in tropical livestock systems. Indigenous breeds such as Landim and Nguni exhibit superior heat tolerance, disease resistance and grazing efficiency (Oloo et al. 2023). However, these breeds are often perceived as less productive than European taurine breeds, which tend to perform well under intensive conditions but experience substantial declines in productivity when exposed to heat stress, nutritional fluctuations and low-input systems typical of Mozambique (Maciel et al. 2013). The performance gap between adapted and non-adapted breeds, particularly under varying management conditions, remains insufficiently documented through quantitative synthesis (Woldeyohannes et al. 2024, Cumbe 2023).

Sustainable management practices have received increasing attention as tools to improve animal comfort, reduce heat stress and enhance the productive capacity of cattle (Cumbe 2023). Practices such as shaded grazing areas, rotational grazing, misting systems and forage conservation have been associated with improved performance and welfare. Nonetheless, empirical evidence from Mozambique remains fragmented and lacks integrated statistical evaluation capable of informing broad policy and production decisions (Rocha et al. 1991, Sitoe 2013, Cumbe et al. 2025). However, adoption of these strategies remains limited, and their broader implications for value chain performance are not yet fully understood.

A comprehensive synthesis of available data is therefore essential. Mozambique currently lacks consolidated evidence quantifying the relative effects of adapted and non-adapted breeds, comparing sustainable and conventional systems, or identifying potential synergistic interactions between genetics and management. Furthermore, there is no systematic assessment of which sustainability interventions are most effective in mitigating heat stress or of the methodological weaknesses that limit scientific progress (Rocha et al. 1991, Cumbula and Taela 2020, Maciel et al. 2013, Dionisio and Syrstad 1990, Carvalheira et al. 1995).

To address these gaps, this study integrates a systematic literature review, a bibliometric assessment and a mixed-effects meta-analysis. Together, these approaches allow a quantitative evaluation of breed performance, system effects, potential synergies and the state of methodological rigor in existing research. By consolidating evidence and estimating predictive models, the study seeks to support decision-making regarding genetic improvement, sustainable intensification and the development of climate-resilient beef production systems in Mozambique. Therefore, this study aimed to (1) systematically review the literature, (2) analyze publication trends, and (3) quantify through meta-analysis the effects of breed adaptation and sustainable systems on cattle weight gain in Mozambique.

## Materials and methods

### Systematic review

The documentary research and study selection process followed the guidelines of the Cochrane Library (Higgins 2023). Data were collected from multiple academic databases, including IEEE Xplore, ISI Web of Science, Scopus, CAB Abstracts, AGRIS (FAO), SciELO, and Google Scholar. These sources were chosen for their comprehensive coverage of agricultural, engineering, and veterinary sciences (Wang and Xu 2025). The dataset included peer-reviewed journal articles, conference proceedings, dissertations, and theses. All database searches were performed on March 15, 2025.

The literature search was conducted using targeted keywords related to the research topic, following the search string presented below. The search focused on Mozambique because the study aimed to provide country-specific evidence for policy and production decisions. However, comparative studies from neighboring countries (South Africa, Zimbabwe, Zambia, Tanzania, Malawi, and Botswana) with similar agro-ecological conditions were included when they provided relevant data for Mozambican contexts.

#### Search string used in all databases

(“beef cattle” OR “cattle production” OR “livestock systems”) AND (“Mozambique” OR “Southern Africa” OR “Sub-Saharan Africa”) AND (“indigenous breeds” OR “adapted breeds” OR “local breeds” OR “tropical cattle”) AND (“sustainable management” OR “grazing management” OR “feeding systems” OR “heat stress”) AND (“average daily gain” OR “weight gain” OR “animal productivity”). The search strategy was designed to balance specificity and sensitivity, allowing the inclusion of studies conducted in Mozambique and comparable agro-ecological regions.

Inclusion criteria required that eligible publications be full-length articles, proceedings, dissertations, or theses published between 1979 and 2024. Studies had to include assessments of beef cattle breeds, production systems, sustainability indicators, or value chain aspects in Mozambique or neighboring countries with comparable conditions. This time frame was selected to capture the period marked by major agricultural policy changes and climate adaptation initiatives in the country (MADER 2020, Pisano 2020, Bessa et al. 2009). To minimize selection bias, two independent reviewers screened the studies in parallel, following Cochrane recommendations. Reviewers worked independently, without communication, and used a standardized list of 48 search terms. Abstracts were initially screened for relevance, followed by full-text assessments to determine whether the studies contained quantitative or qualitative data relevant to the research questions. Studies were excluded if they lacked essential information such as breed type, production system description, sustainability indicators, economic data, or value chain parameters (Firdaus et al. 2023, Vaz et al. 2017). All eligible data were compiled into a structured dataset. For each variable, the reported mean, standard deviation (SD) or coefficient of variation (CV), and sample size (n) were extracted to support subsequent statistical analysis.

### Bibliometric review

Bibliometric Literature Reviews (BLRs) are designed to examine, identify, and evaluate trends, publication patterns, research impact, and the evolution of a specific field of study over time. They provide a comprehensive overview of the current state of knowledge within a discipline, highlighting research gaps, predominant areas of focus, temporal changes, and influential contributors to the field (Hallinger and Kovačević 2023, Montazeri et al. 2023, Hoang 2025, Büchter et al. 2020). In this study, a BLR approach was applied to generate a word cloud based on keywords extracted from the selected publications. In addition, graphical analyses were produced to represent variables such as year of publication, geographical distribution of studies, cattle breeds investigated, production systems described, sustainability practices reported, and value chain segments analyzed. These variables were evaluated across all included studies. This analytical process was systematically applied to the entire database generated by the SLR, following the methodologies previously reported in related studies (Firdaus et al. 2023). The word clouds were generated using the open-acess platform WordClouds.com, and the other representations were developed in Microsoft Excel^®^.

### Definitions of key variables

To ensure clarity and reproducibility, the following definitions were adopted throughout this study. **Breed type**: Adapted breeds were defined as indigenous cattle breeds (Landim, Nguni, and Angoni) that have evolved in tropical environments and possess physiological adaptations to heat stress, nutritional fluctuations, and local diseases. Non-adapted breeds were defined as European taurine breeds (i.e., Holstein, Angus, and Hereford) that were introduced to Mozambique and typically require higher input conditions. **Production system type**: Sustainable systems were defined as management practices that include at least two of the following interventions: shading, rotational grazing, improved feeding (supplementation), misting systems, or forage conservation. Traditional (conventional) systems were defined as extensive management with no or minimal intervention beyond basic herd management. **Weight gain measurement**: Daily weight gain (kg/day) was calculated as the total weight gain divided by the number of days between initial and final weighing. The evaluation period varied across studies but was consistently reported as average daily gain (ADG) over periods ranging from 90 to 365 days. **Sustainability level**: A production system was classified as “sustainable” if it incorporated management practices aimed at reducing environmental impact, improving animal welfare, or enhancing resource use efficiency, as reported by the original study authors.

### Meta-analysis

For the meta-analysis used to adjust regression models, the following input variables were defined: breed type (categorical: adapted vs. non-adapted) and sustainability level (categorical: sustainable vs. conventional). The output variables included weight gain (kg/day), reproductive rate (%), and heat stress reduction (%). To integrate data from different studies, a meta-analysis was performed following the methodology described by St-Pierre (2007), which has been widely adopted in recent research (Stanley and Doucouliagos 2015, Moreno et al. 2012, Domínguez Islas and Rice 2018). Because the studies differed in sample sizes and precision, a weighted regression approach was employed (Moreno et al. 2012, Domínguez Islas and Rice 2018). Each study was weighted according to the inverse of its variance, ensuring that studies with higher statistical precision exerted greater influence on the model estimates. The weight assigned to each observation was calculated as the inverse of the squared standard error (SE²). The original meta-analysis approach by(St-Pierre 2007) was adapted due to missing data in some studies. To address missing dispersion measures in some studies, a data harmonization approach was applied. When standard errors were not directly reported, they were estimated from available statistics such as standard deviation (SD), coefficient of variation (CV), and sample size (n), following established meta-analytical procedures. This approach allowed the inclusion of relevant studies without excluding incomplete datasets, while preserving the original reported mean values. Although this procedure follows established practices in meta-analysis, it may introduce additional uncertainty and is therefore acknowledged as a limitation of the study. In the regression model, breed and production system were treated as fixed effects, while study-specific differences were modeled as random effects. The general structure of the model is shown in Eq. 1.

$$\:{Y}_{ij}={\beta\:}_{0}+{\beta\:}_{1}\cdot\:{X}_{1j}+{\beta\:}_{2}\cdot\:{X}_{2j}+{S}_{i}+{\epsilon}_{ij}$$

Where: Y is the productivity response variable (weight gain), β₀ is the intercept, β₁ is the effect of breed type, β₂ is the effect of sustainability level, X₁ is breed type (1 = adapted, 0 = non-adapted), X₂ is system type (1 = sustainable, 0 = conventional), S_i_ is the random effect of study i, and ε_i_ⱼ ~ N(0, σ²) is the residual error.

To evaluate the consistency among studies, statistical heterogeneity was assessed using Cochran’s Q statistic and the I² index. The Q statistic tests whether the observed variability among study estimates exceeds the variability expected by sampling error alone, while the I² statistic quantifies the proportion of total variability attributable to real differences among studies. Values of I² below 25% were interpreted as low heterogeneity, values between 25% and 50% as moderate heterogeneity, and values above 50% as high heterogeneity. The models were fitted using the lme function from the nlme package in R version 4.3.1 (Core 2025). Statistical significance was assessed at the 5% probability level. Assessment of risk of bias: A formal risk of bias assessment using standardized tools (e.g., ROBINS-I or Cochrane Risk of Bias) was not performed. However, heterogeneity across studies was evaluated using Cochran’s Q statistic and the I² index, as described above. The lack of a formal risk of bias assessment is acknowledged as a limitation of this study (Section “Limitations of the study”).

### Model verification

Data from studies that reported mean values for weight gain, reproductive rate, and heat stress reduction, but were not used in developing the regression model, were employed for model validation. Validating the model with an independent dataset is essential to assess its generalizability, detect potential overfitting, and ensure robustness. Weight gain was estimated using observed values of breed type and sustainability level. To assess the accuracy of the model, the root mean square error (RMSE) was computed, which measures the average magnitude of prediction errors between observed and estimated values. Lower RMSE values indicate better predictive accuracy and improved model performance.

### Application of the model

The predictive model developed through the meta-analysis was applied to estimate daily weight gain under four production scenarios defined by combinations of breed type (adapted vs. non-adapted) and production system (sustainable vs. traditional/conventional). Economic viability thresholds were defined a priori based on published benchmarks. Beef cattle production was considered economically viable when daily weight gain exceeded 0.5 kg/day under smallholder conditions and 0.8 kg/day under commercial systems, following the criteria proposed by Jordaan et al. (2021), and Timberlake (Jonathan 2025).

## Results

### Systematic review results

**Fig. 1:**
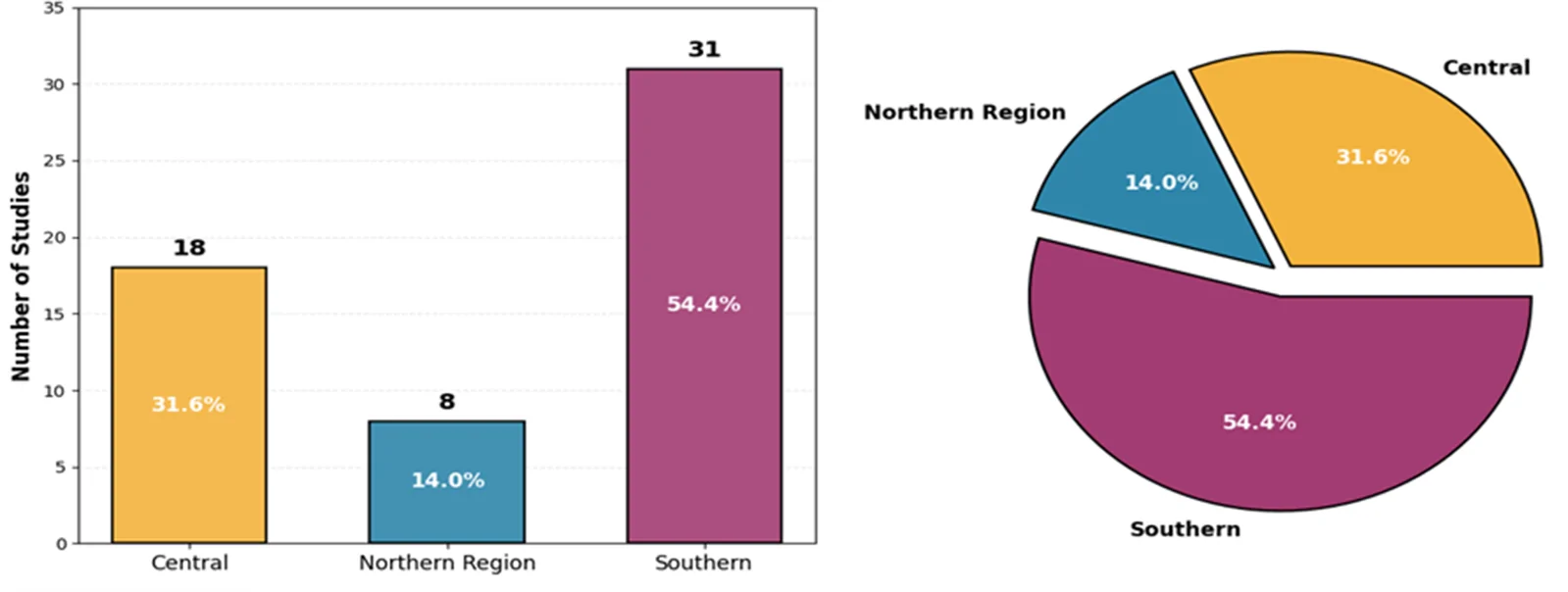
Geographical distribution of beef cattle studies across regions of Mozambique

The systematic review resulted in the identification and selection of 57 scientific publications addressing beef cattle production in Mozambique. The final database comprised peer-reviewed journal articles, theses, dissertations, and conference proceedings published between 1979 and 2024, encompassing both early foundational research and more recent scientific developments in the sector. Although the search strategy included international databases, the majority of selected studies were conducted within Mozambique, with a limited number incorporating comparative analyses with neighboring countries under similar agro-ecological conditions. The geographical distribution of the studies shows a clear concentration in the southern and central regions of the country. Research carried out in provinces such as Maputo, Gaza, Inhambane, Manica, and Sofala predominates, reflecting higher cattle densities, the historical presence of research institutions, and better logistical access. In contrast, the northern region is consistently under-represented, despite its relevance for extensive and smallholder cattle systems. This spatial imbalance highlights an important research gap, particularly concerning productivity, adaptation, and sustainability under the climatic and socio-economic conditions of northern Mozambique (Fig. 1).

Across the reviewed literature, dominant research themes included the characterization of cattle production systems, growth and reproductive performance of indigenous breeds (especially Landim, Nguni, and Angoni), comparisons with non-adapted or exotic breeds, and management practices under extensive and smallholder conditions. More recent studies increasingly focused on climate change impacts, genetic diversity, heat stress, and sustainability-oriented interventions, indicating a progressive shift in research priorities toward resilience and adaptation strategies. From the total set of reviewed publications, 15 studies met the criteria for quantitative synthesis and meta-analysis, providing sufficient information on mean daily weight gain, variability measures, and production system characteristics. These studies formed the analytical dataset used to estimate the effects of breed adaptation and production system on cattle performance. The PRISMA 2020 flow diagram summarizing the search and selection process is provided as supplementary material. The main descriptive characteristics of these studies, including sample size, breed type, production system, weight gain parameters, and study region, are summarized in Table 1.

**Table 1:** Descriptive characteristics of the studies included in the meta-analysis

Study	e	*n*	Breed type	System type	WG mean	WG SD	WG SE	Region
(Carvalheira et al. 1995, Joaquim et al. 2024)	1	45	Adapted	Traditional	0.42	0.15	0.022	South
(Cumbe et al. 2021, Escrivão et al. 2012)	2	32	Adapted	Sustainable	0.68	0.18	0.032	Central
(Joaquim et al. 2024, King et al. 2021)	3	28	Non adapted	Sustainable	0.51	0.22	0.042	South
(Vernooij et al. 2016, Madilindi et al. 2020)	4	56	Non adapted	Traditional	0.31	0.12	0.016	North
(Dionisio and Syrstad 1990)	5	38	Adapted	Traditional	0.59	0.16	0.026	Central
(Paula Pimentel 2025, Maciel et al. 2012)	6	41	Adapted	Sustainable	0.74	0.19	0.030	South
(Otte and Chilonda 2003)	7	29	Non adapted	Sustainable	0.48	0.21	0.039	Central
(Rocha et al. 1991, Macuvele et al. 2025)	8	34	Non adapted	Traditional	0.29	0.14	0.024	South
(Maciel et al. 2012)	9	47	Adapted	Traditional	0.61	0.17	0.025	North
(Cumbe et al. 2021)	10	39	Adapted	Sustainable	0.79	0.20	0.032	Central
(Mccarthy et al. 2023)	11	31	Non adapted	Traditional	0.33	0.13	0.023	South
(Paula Pimentel 2025)	12	42	Adapted	Sustainable	0.72	0.18	0.028	Central
(Vernooij et al. 2016)	13	36	Non adapted	Sustainable	0.53	0.23	0.038	North
(Carvalheira et al. 1995)	14	44	Adapted	Traditional	0.57	0.15	0.023	South
(Mccarthy et al. 2023)	15	36	Non-Adapted	Traditional	0.28	0.11	0.019	Central

Where: e - number given to the study, n - sample number, Breed_type - adapted (Landim, Nguni) vs. non-adapted (European purebreds), System_type - sustainable (shading, misting, rotational grazing) vs. traditional (conventional), WG_mean - average weight gain (kg/day), WG_SD - standard deviation of weight gain, WG_SE - standard error of weight gain, Region - study location in Mozambique

Footnote to Table 1: Adapted breeds include Landim, Nguni, and Angoni. Non-adapted breeds include European taurine breeds (i.e., Holstein, Angus, and Hereford). Sustainable systems include practices such as shading, rotational grazing, improved feeding, misting systems, or forage conservation. Traditional (conventional) systems refer to extensive management with no or minimal intervention. Weight gain (WG) is reported as average daily gain (kg/day) over periods ranging from 90 to 365 days. Important limitation: None of the included studies directly compared adapted and non-adapted breeds under identical management conditions. Therefore, the observed effects may be partially confounded by differences in management, environment, and other study-specific factors. This limitation is addressed in Section “Limitations of the study”

### Bibliometric review

**Fig. 2:**
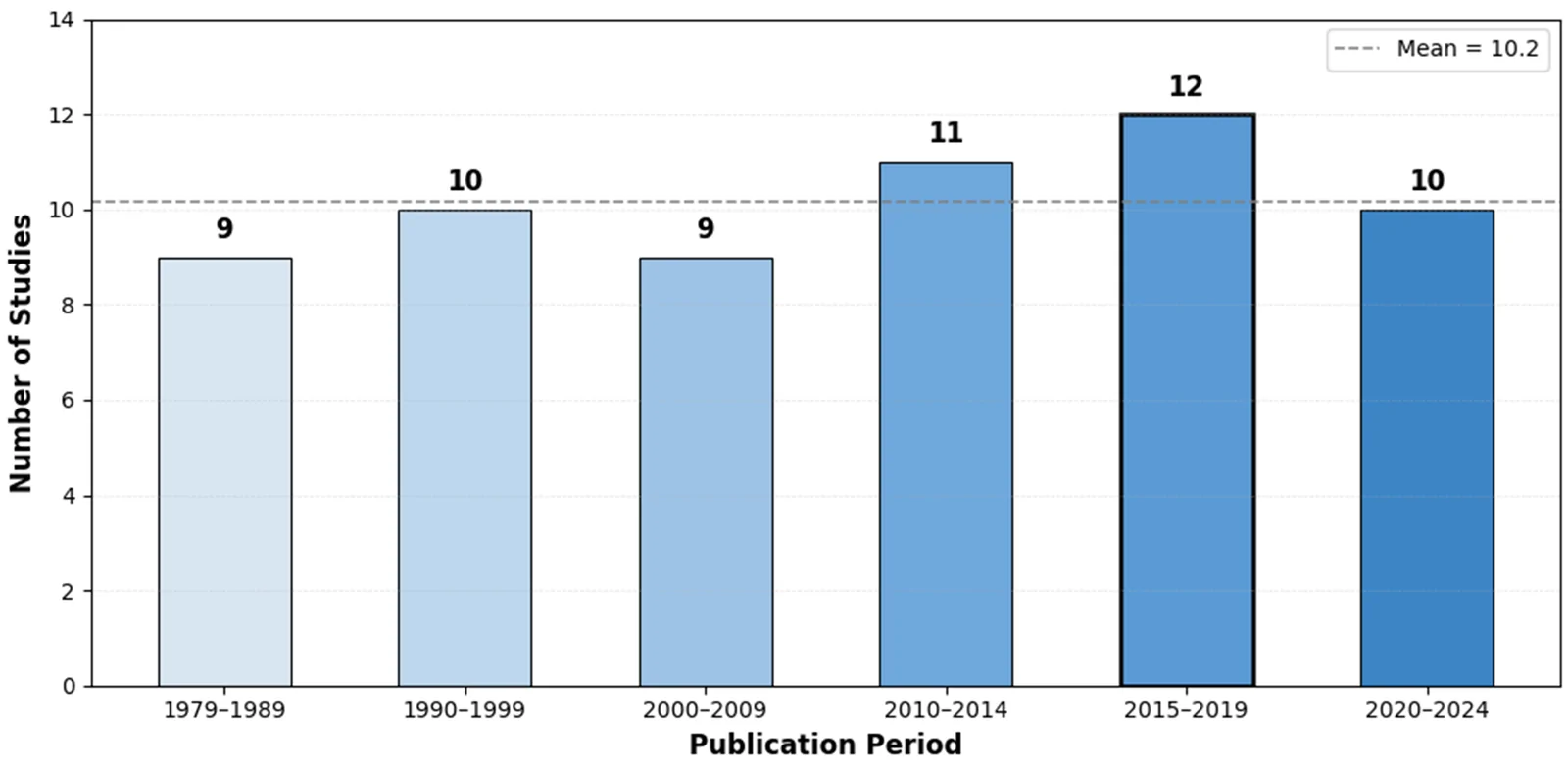
Temporal distribution of scientific publications on beef cattle production in Mozambique (1979–2024). The figure shows three distinct phases: Phase 1 (1979–1999) - foundational studies on indigenous breeds; Phase 2 (2000–2009) - reduced publication activity; Phase 3 (2010–2024) - increased focus on climate change, sustainability, and smallholder systems. To complement the bibliometric analysis, a keyword frequency assessment was conducted based on titles, abstracts, and author keywords

The bibliometric analysis provided insights into publication trends, geographical focus, and thematic evolution of beef cattle research in Mozambique. The 57 studies included in the review were published between 1979 and 2024. Figure 2 illustrates the three main phases of research development: an initial period focused on breed characterization and extensive systems (1979–1999), a phase of reduced publication activity (2000–2009), and a recent period marked by increased research output emphasizing climate change, sustainability, and productivity in smallholder systems (2010–2024).

**Fig. 3:**
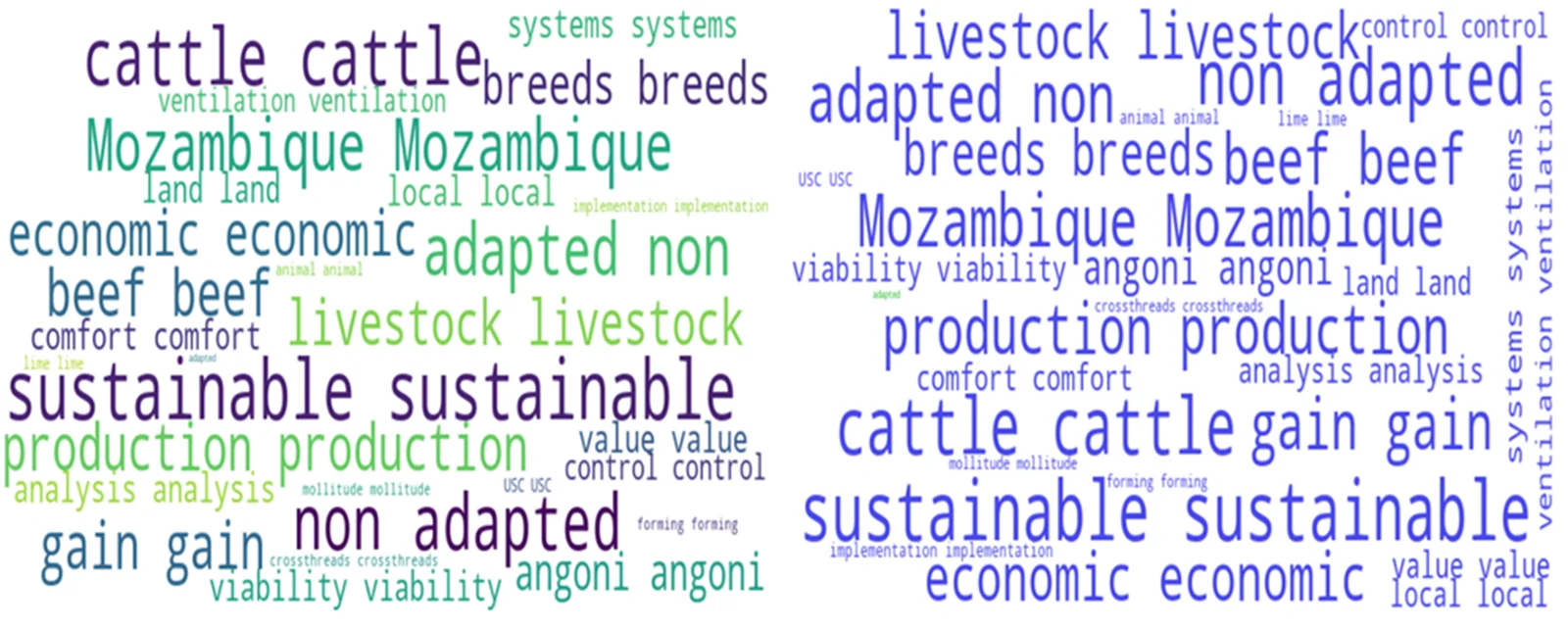
Word cloud representing the most frequent keywords in Mozambican beef cattle research. Larger font sizes indicate higher frequency of occurrence. Colors are used for visual distinction only and do not represent thematic categories

Figure 3 presents a word cloud where the size of each word reflects its frequency in the literature. The word cloud is organized with the most frequent terms (“Mozambique”, “cattle”, “production”, “breeds”) appearing in larger font sizes. The color variation is aesthetic only and does not represent different analytical categories (wordcloud 2025).

### Meta-analysis of weight gain

**Fig. 4:**
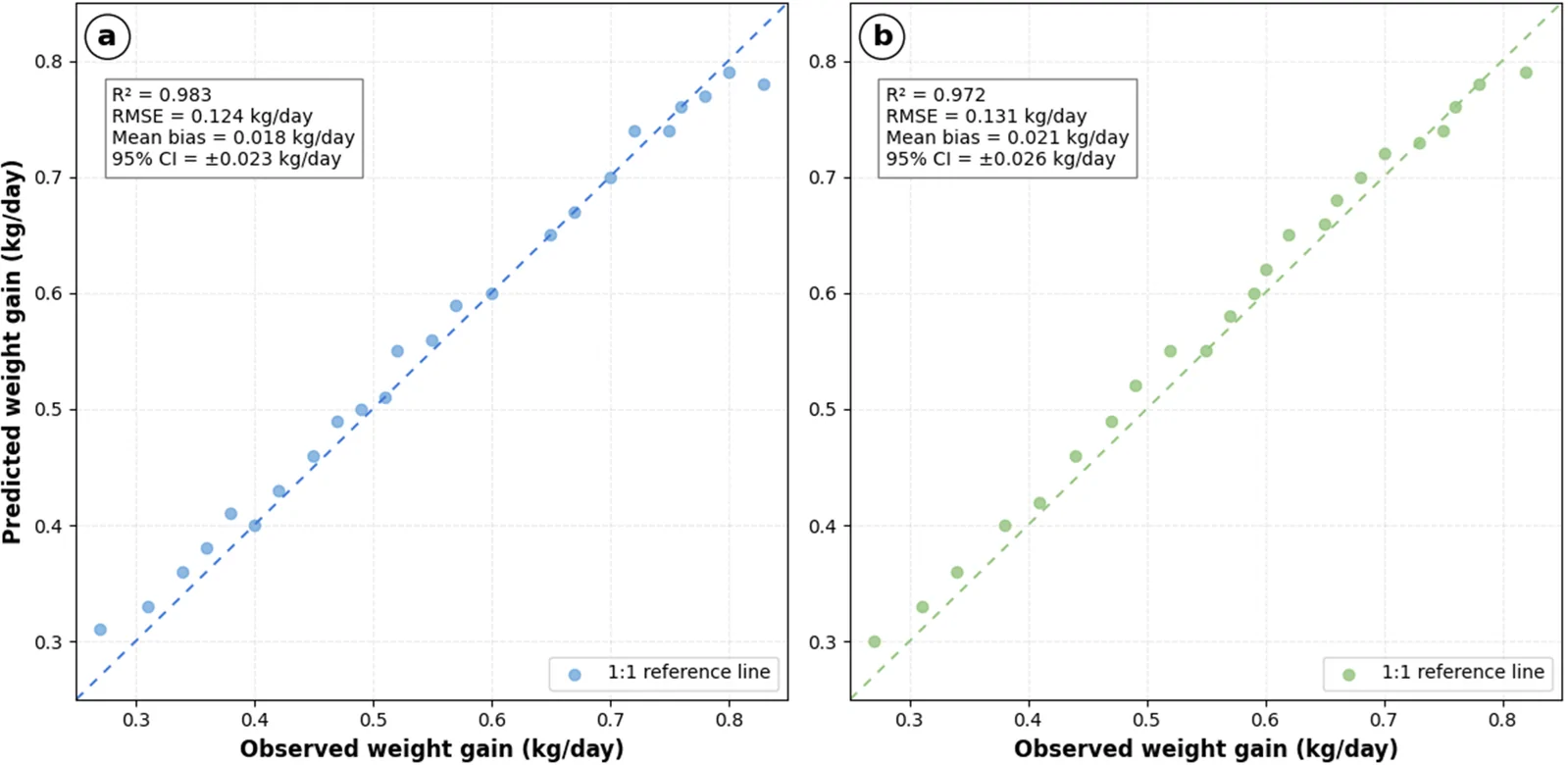
Relationship between observed and predicted daily weight gain values obtained from (**a**) model calibration using the meta-analysis dataset and (**b**) model validation. The dashed line represents the 1:1 relationship between observed and predicted values

Fifteen studies from the systematic review met the inclusion criteria for the meta-analysis, as they reported mean daily weight gain along with measures of variability (SD, CV, or SE) and sample size. These studies encompassed both adapted breeds (Landim and Nguni) and non-adapted breeds (European taurine breeds, purebreds or crossbreeds), evaluated under traditional (conventional) and sustainable production systems across Mozambique’s three main regions. The variability observed across studies was moderate, with a standard deviation of 0.184 kg/day for weight gain, indicating heterogeneity in management conditions, environmental factors, and genetic backgrounds. Mixed-effects meta-regression analysis showed that breed adaptation and sustainability level of the production system had statistically significant positive effects on daily weight gain (*p* < 0.05). In contrast, models developed for reproductive rate and heat stress reduction were not statistically significant and were therefore not retained for further analysis. Potential reasons for non-significance: The lack of significance for reproductive rate and heat stress reduction may be attributed to insufficient sample sizes, inconsistent measurement methods across studies, or the absence of direct comparisons under standardized conditions. The final meta-analytical model generated a predictive Eq. 2, as illustrated in Fig. 4, with breed type and production system treated as fixed effects and study identity as a random effect.

$$\begin{aligned}\:Weight\:gain\:\left(\frac{kg}{day}\right)&=\:0.312\:+\:0.278\cr&\:\times\:\:Breed\_adapted\:\cr&+\:0.194\:\cr&\times\:\:System\_sustainable\end{aligned}$$

Breed_adapted = 1 for adapted breeds (Landim, Nguni, Angoni) and 0 for non-adapted breeds (European taurine breeds), and System_sustainable = 1 for sustainable systems (shading, rotational grazing, improved feeding, misting, or forage conservation) and 0 for traditional/conventional systems. Among the studies included in model development, 72.4% were conducted under smallholder conditions, while 27.6% represented commercial or semi-commercial systems, reinforcing the relevance of the model for smallholder-based production in Mozambique.

### Model validation

Model validation was conducted using independent studies from the systematic review that reported mean values of weight gain but lacked sufficient dispersion measures to be included in the meta-regression. Observed values from these studies were compared with predicted values generated by the model. The validation results indicate a strong agreement between observed and predicted weight gain values. The mean standard error (0.06 kg/day) and mean bias (0.018 kg/day) were low, suggesting minimal systematic error. As illustrated in Fig. 4b, observed and predicted values closely follow the 1:1 reference line, confirming the robustness and generalizability of the model across different regions, breed types, and production systems. The statistical performance of the model is summarized in Table 2, which presents the main validation metrics. The coefficient of determination (R² = 0.983) indicates excellent explanatory power, while the RMSE of 0.124 kg/day reflects high predictive accuracy. The mean standard error (0.06 kg/day) and mean bias (0.018 kg/day) were low, suggesting minimal systematic error. Based on the mean SE, the estimated 95% confidence interval (± 0.023 kg/day) falls within acceptable limits for practical application.

**Table 2:** Statistical performance metrics of the meta-analytical model

Metric	Value
R²	0.983
RMSE	0.124 kg/day
Mean SE	0.06
Mean bias	0.018 kg/day
95% CI	± 0.023 kg/day

### Application of the model

**Fig. 5:**
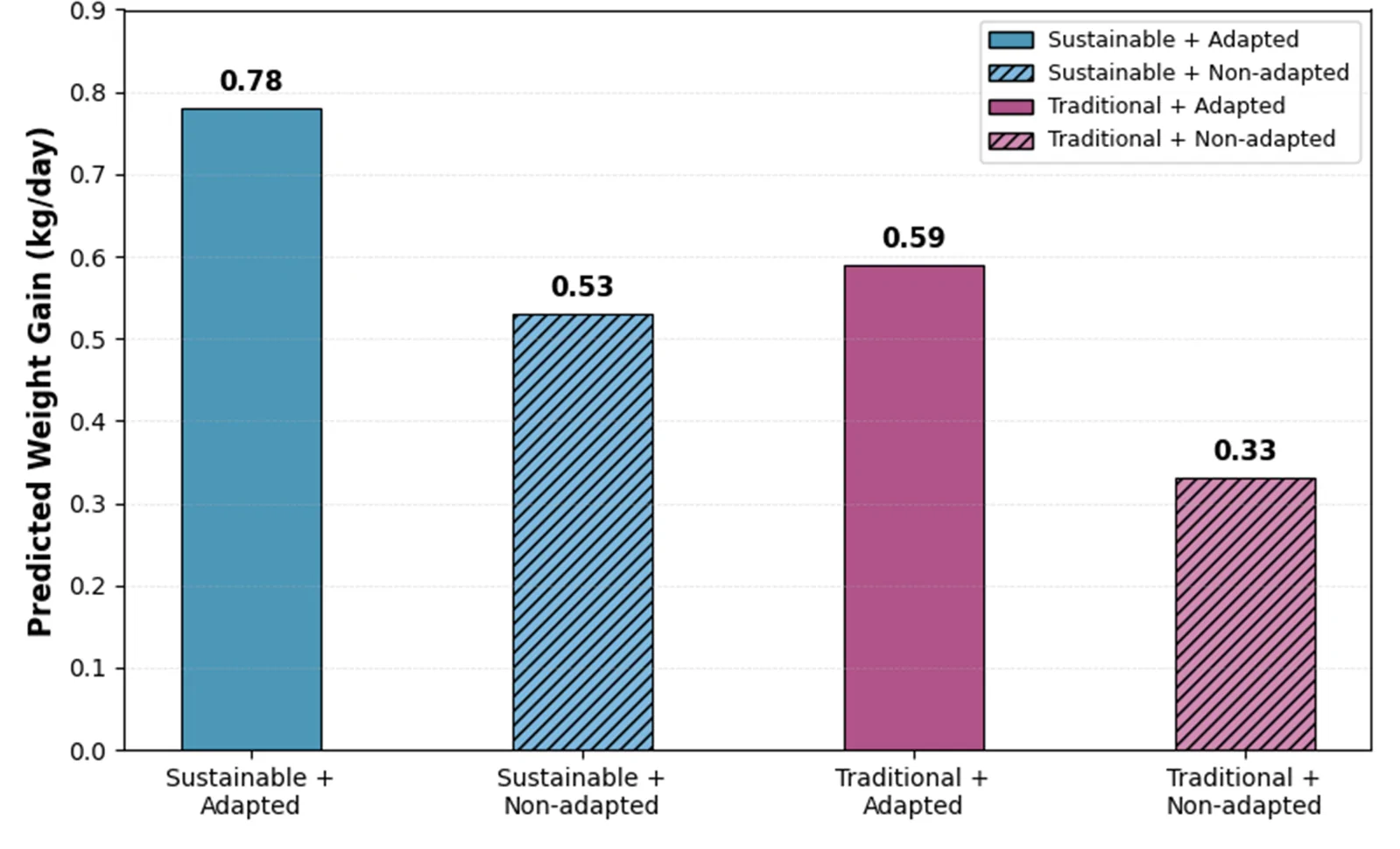
Predicted daily weight gain across breed and production system scenarios

The validated model was applied to estimate daily weight gain under four production scenarios defined by the interaction between breed type and production system (Fig. 5). These scenarios represent practical conditions observed in Mozambican cattle production systems. Non-adapted + Traditional system: This scenario represents European taurine breeds (e.g., Holstein, Angus, Hereford) managed under extensive smallholder systems characterized by low technological input, minimal supplementation, and limited environmental control. Adapted + Traditional system: This scenario includes indigenous breeds (Landim, Nguni, Angoni) managed under similar extensive conditions. These breeds are naturally adapted to local climatic stressors, including heat and feed variability. Non-adapted + Sustainable system: In this scenario, non-adapted breeds are managed using improved practices such as shading, rotational grazing, and enhanced feeding strategies aimed at reducing heat stress and improving productivity. Adapted + Sustainable system: This scenario combines locally adapted breeds with sustainable management practices, representing the most optimized production condition evaluated in this study. The predicted values were compared against established economic viability thresholds of 0.5 kg/day for smallholder systems and 0.8 kg/day for commercial systems. Under traditional systems with non-adapted breeds, the predicted daily weight gain was 0.31 kg/day, substantially below the minimum threshold for economic viability. This result reflects the limited adaptability of these breeds under low-input tropical conditions. In contrast, adapted breeds under traditional systems achieved a predicted gain of 0.59 kg/day, exceeding the smallholder viability threshold. This indicates that genetic adaptation alone can significantly improve productivity, even in the absence of improved management. When sustainable practices were adopted, performance improved across both breed types. Non-adapted breeds under sustainable systems reached 0.51 kg/day, representing marginal economic viability for smallholders, suggesting that improved management can partially compensate for limited genetic adaptation. The highest predicted performance was observed for adapted breeds under sustainable systems, with an estimated daily weight gain of 0.78 kg/day. As shown in Fig. 5, this scenario consistently outperforms all others, highlighting the synergistic effect between genetic adaptation and sustainable management practices.

## Discussion

The results of this study provide a consolidated quantitative synthesis of beef cattle productivity in Mozambique, integrating evidence from 57 publications and a meta-analytical framework based on 15 studies with comparable productivity indicators. The findings reinforce the central role of genetic adaptation and management practices in determining cattle performance under Mozambican conditions and align closely with both classical and recent literature on indigenous cattle systems in southern Africa.

The systematic review revealed a strong geographical concentration of studies in southern and central Mozambique, a pattern also reported by Rocha et al.(1991) and Sitoe (2013), which emphasize the historical focus of research institutions in these regions. The persistent under-representation of northern Mozambique observed in this review confirms earlier concerns raised by Vernooij et al. (2016), indicating a critical knowledge gap regarding productivity, adaptation, and sustainability in northern agro-ecological zones. The bibliometric analysis demonstrates a clear evolution of research priorities over time. Early studies (Carvalheira et al. 1995), focused primarily on breed characterization, growth performance, and reproductive traits under extensive conditions. This descriptive phase laid the foundation for understanding the biological potential of indigenous breeds such as Landim, Nguni, and Angoni. From 2010 onward, increasing emphasis on climate change, sustainability, and genetic diversity were observed in studies such as McCarthy et al. (2023), and other recent works, reflecting a broader regional and global shift toward resilience-oriented livestock research.

The meta-analysis confirms that adapted breeds exhibit significantly higher daily weight gain than non-adapted breeds under Mozambican production conditions. Heat stress represents one of the most important constraints affecting cattle productivity in tropical environments. Indigenous breeds such as Nguni and Landim have evolved physiological adaptations that improve thermoregulation, including higher sweating capacity, lower metabolic heat production, and better tolerance to seasonal fluctuations in feed availability (Maciel et al. 2013, Dionisio and Syrstad 1990), and (Carvalheira et al. 1995). These characteristics enable them to maintain relatively stable productivity under environmental conditions in which exotic taurine breeds frequently experience severe declines in growth performance and reproductive efficiency. Similarly, the positive effect of sustainable management practices on weight gain corroborates results from Pimentel et al.(2025) and Cumbe et al. (2021), which demonstrated that relatively simple interventions, such as improved feeding, shade provision, and better grazing management, can substantially enhance productivity. The present study extends these findings by quantifying the average magnitude of this effect across multiple studies, demonstrating that sustainability-oriented practices improve performance regardless of breed type.

The combined effect of adapted genetics and sustainable management resulted in the highest predicted daily weight gain (0.78 kg/day), approaching the threshold for commercial viability. This synergistic interaction aligns with conclusions drawn by Easter et al. (2018), who argue that genetic resources alone are insufficient unless complemented by appropriate management strategies. Likewise, Mwai et al. (2015) emphasize that the productive potential of indigenous breeds can only be fully realized when management constraints are addressed. From a policy and development perspective, the findings support recommendations (Cumbula and Taela 2020) (Cumbe et al. 2021), which advocate prioritizing indigenous breeds and context-specific management improvements rather than relying on poorly adapted exotic genetics. The results also underscore the importance of integrating sustainability interventions into smallholder systems, where most Mozambican cattle production occurs, as emphasized by (Koide and Tinga 2021) and (Koide and Tinga 2017). Overall, this study contributes to the existing literature by moving beyond individual case studies and providing a quantitative synthesis of breed and management effects on beef cattle productivity in Mozambique. By combining systematic review, bibliometric analysis, and meta-regression, it offers an evidence-based framework to guide genetic improvement, sustainable intensification, and climate adaptation strategies. The results reinforce that aligning adapted genetics with sustainable management practices is the most effective pathway to improving productivity, resilience, and economic viability of beef cattle systems in Mozambique.

## Conclusions

This study provides the first integrated synthesis combining systematic review, bibliometric analysis, and meta-analytical modeling to evaluate beef cattle production performance in Mozambique. The results demonstrate that genetic adaptation and production system sustainability are the primary drivers of daily weight gain under Mozambican conditions. Adapted breeds such as Landim, Nguni, and Angoni consistently outperform non-adapted breeds in terms of growth performance, particularly under extensive and smallholder systems. Sustainable management practices further enhance productivity across breed types, with the greatest gains observed when adapted genetics are combined with improved management strategies. The meta-analytical model shows strong predictive performance (R² = 0.983, RMSE = 0.124 kg/day) and confirms that this combination approaches commercial viability thresholds, even under predominantly smallholder conditions. The findings highlight the importance of preserving and promoting indigenous genetic resources while simultaneously investing in sustainable intensification strategies. From a policy and development perspective, the results support interventions that prioritize adapted breeds, climate-resilient management practices, and region-specific production strategies. Future research should focus on under-represented northern regions and on integrating economic and environmental indicators to further refine decision-making tools for Mozambique’s beef cattle sector.

## Limitations of the study

Despite the rigorous methodological framework applied, several limitations should be acknowledged. First, the number of studies providing complete statistical information suitable for meta-analysis was relatively limited (*n* = 15). Although this is consistent with the availability of quantitative data in the literature, it may restrict the statistical power and generalizability of the model. Second, substantial heterogeneity was observed across studies, reflecting differences in experimental design, feeding systems, environmental conditions, and measurement periods. While this variability is inherent to studies conducted under real-world conditions, it may introduce uncertainty not fully captured by the model, despite the use of a mixed-effects approach. Third, a data harmonization approach was required to address missing dispersion measures in some studies. In these cases, variance parameters were estimated from available statistics (e.g., SD, CV, and sample size). Although this procedure follows established meta-analytical practices, it may introduce additional uncertainty into the model estimates. Fourth, none of the included studies directly compared adapted and non-adapted breeds under identical management conditions. As a result, the observed effects of breed type may be partially confounded by differences in management practices, environmental conditions, and study-specific factors. Fifth, a formal risk of bias assessment using standardized tools was not conducted due to the heterogeneity of study designs. Instead, a qualitative evaluation was performed, considering study characteristics and potential sources of bias. Nevertheless, this represents a limitation in terms of methodological rigor. Sixth, several potentially relevant confounding variables, such as detailed nutritional composition, disease prevalence, and microclimatic conditions, were not consistently reported across studies and therefore could not be incorporated into the analysis. Finally, a geographical imbalance was identified, with a clear under-representation of studies from northern Mozambique. This limits the extrapolation of results to that region, where production systems and environmental conditions may differ substantially. Despite these limitations, the use of a mixed-effects meta-regression framework allows for the explicit incorporation of between-study variability, increasing the robustness and reliability of the results. Overall, the findings should be interpreted as evidence-based trends rather than precise estimates, providing a solid foundation for future research and decision-making.

## Data Availability

All data generated or analysed during this study are included in this published article and its supplementary information files.

## Declarations

### Dual publication

The authors confirm that this manuscript is original, has not been published previously, and is not under consideration for publication elsewhere.

## References

[CR22] Bessa I, Pinheiro I, Matola M, Dzama K, Rocha A, Alexandrino P (2009) Genetic diversity and relationships among indigenous Mozambican cattle breeds. S Afr J Anim Sci 39:61–72. 10.4314/SAJAS.V39I1.43548

[CR28] Büchter RB, Weise A, Pieper D (2020) Development, testing and use of data extraction forms in systematic reviews: a review of methodological guidance. BMC Med Res Methodol 20:1–14. 10.1186/S12874-020-01143-3/TABLES/3 PMC7574308 33076832

[CR4] Carvalheira JG, Blake RW, Pollak EJ, Van Soest PJ (1995) Comparison of Landim and Africander cattle in southern Mozambique: I. Body weights and growth. J Anim Sci 73:3519–3526. 10.2527/1995.73123519X 8655424

[CR50] Carvalheira JG, Blake RW, Pollak EJ, Van Soest PJ (1995) Comparison of Landim and Africander cattle in southern Mozambique: I. Body weights and growth. J Anim Sci 73:3519–3526. 10.2527/1995.73123519X 8655424

[CR17] Carvalheira JG, Blake RW, Pollak EJ, Van Soest PJ (1995) Comparison of Landim and Africander cattle in southern Mozambique: II. Female fertility, reproduction, and beef offtake. J Anim Sci 73:3527–3533. 10.2527/1995.73123527X 8655425

[CR36] Carvalheira JG, Blake RW, Pollak EJ, Van Soest PJ (1995) Comparison of Landim and Africander cattle in southern Mozambique: II. Female fertility, reproduction, and beef offtake. J Anim Sci 73:3527–3533. 10.2527/1995.73123527X 8655425

[CR53] Carvalheira JG, Blake RW, Pollak EJ, Van Soest PJ (1995) Comparison of Landim and Africander cattle in southern Mozambique: II. Female fertility, reproduction, and beef offtake. J Anim Sci 73:3527–3533. 10.2527/1995.73123527X 8655425

[CR10] Cumbe TA (2023) Análise de fatores associados à dinâmica do desenvolvimento dos sistemas de produção de bovinos: o caso de Moçambique

[CR38] Cumbe TA, Sessim AG, López-González FA, Zago D, Alforma AMP, Barcellos JOJ (2021) Bioeconomic evaluation of feedings strategies in the yearling beef cattle system in Mozambique. Livest Sci 247:104466. 10.1016/J.LIVSCI.2021.104466

[CR56] Cumbe TA, Sessim AG, López-González FA, Zago D, Alforma AMP, Barcellos JOJ (2021) Bioeconomic evaluation of feedings strategies in the yearling beef cattle system in Mozambique. Livest Sci 247:104466. 10.1016/J.LIVSCI.2021.104466

[CR13] Cumbe TA, Cunguara BA, McManus CM, Alforma AMP, Barcellos JOJ (2025) An understanding of the motivations that influence the beef cattle production systems adopted by farmers in central Mozambique. PLoS ONE 20:e0325929. 10.1371/JOURNAL.PONE.0325929 PMC12173388 40526609

[CR14] Cumbula D, Taela M (2020) Animal genetic resources (AnGR) in Mozambique. IOP Conf Ser Earth Environ Sci 482:012045. 10.1088/1755-1315/482/1/012045

[CR16] Dionisio AC, Syrstad O (1990) Productivity of Nguni and Africander cattle in Mozambique. Livest Prod Sci 24:29–36. 10.1016/0301-6226(90)90029-6

[CR42] Dionisio AC, Syrstad O (1990) Productivity of Nguni and Africander cattle in Mozambique. Livest Prod Sci 24:29–36. 10.1016/0301-6226(90)90029-6

[CR32] Domínguez Islas C, Rice KM (2018) Addressing the estimation of standard errors in fixed effects meta-analysis. Stat Med 37:1788. 10.1002/SIM.7625 PMC6001579 29574827

[CR54] Easter TS, Killion AK, Carter NH (2018) Climate change, cattle, and the challenge of sustainability in a telecoupled system in Africa. Ecology and Society, Published Online: Jan 22, 2018. 23. 10.5751/ES-09872-230110

[CR39] Escrivão RJA, Webb EC, Garcês AP, de JT, Grimbeek RJ (2012) Effects of 48-hour calf withdrawal on conception rates of Bos indicus cows and calf weaning weights in extensive production systems. Trop Anim Health Prod 44:1779–1782. 10.1007/S11250-012-0137-X 22528530

[CR23] Firdaus F, Atmoko BA, Baliarti E, Widi TSM, Maharani D, Panjono P (2023) The meta-analysis of beef cattle body weight prediction using body measurement approach with breed, sex, and age categories. J Adv Vet Anim Res 10:630. 10.5455/JAVAR.2023.J718 PMC10868685 38370885

[CR25] Hallinger P, Kovačević J (2023) Applying bibliometric review methods in education: rationale, definitions, analytical techniques, and illustrations. Int Encyclopedia Education: Fourth Ed 546–556. 10.1016/B978-0-12-818630-5.05070-3

[CR18] Higgins J & W V. Cochrane handbook for systematic reviews of interventions version 6.4 2023

[CR27] Hoang AD (2025) Evaluating Bibliometrics Reviews: A Practical Guide for Peer Review and Critical Reading. Eval Rev. 10.1177/0193841X251336839 PMC12476466 40251511

[CR37] Joaquim LA, Changule AP, da Glória Taela M, Novela M, Pinto SC, Bila CG (2024) The generation interval and season of birth do not affect age at first calving, birth weight and calving interval of Mozambican Angoni cattle. Trop Anim Health Prod 56. 10.1007/S11250-024-04030-X 38801479

[CR35] Jonathan Timberlake (2025) Inventory of feed resources for small-scale livestock production in Mozambique 2020. https://www.fao.org/4/x5548e/x5548e0a.htm

[CR34] Jordaan FJ, Neser FWC, Maiwashe A, King Z, Scholtz MM (2021) The environmental impact of changes in cow productivity and its component traits in South Africa’s Landrace Beef Breeds. Front Anim Sci 2. 10.3389/fanim.2021.743229

[CR40] King FJM, Banga CB, Visser C (2021) Genetic diversity and population structure of three native cattle populations in Mozambique. Trop Anim Health Prod 53. 10.1007/S11250-021-02562-0 33438116

[CR5] King FJM, Visser C, Banga C (2022) Genetic characterization of Mozambican Nguni cattle and their relationship with indigenous populations of South Africa. Livest Sci 264:105044. 10.1016/J.LIVSCI.2022.105044

[CR58] Koide J, Tinga BI (2017) Whole-farm economic analyses of smallholder crop-dairy systems in southern Mozambique 97–106

[CR57] Koide J, Tinga BI (2021) Viability of smallholder dairy cattle management and its intensification strategies based on whole-farm analyses in southern Mozambique. Trop Anim Health Prod 53:130. 10.1007/S11250-020-02547-5 33462732

[CR2] Macia S, Filimone C (2024) The demand for imported meat in Mozambique: An Application of Almost Ideal Demand System. SSRN Electron J. 10.2139/SSRN.5080879

[CR44] Maciel SMA, Amimo J, Martins M, Mwai AO, Scholtz MM, Neser FWC (2012) Factors influencing reproductive performance of cows from different Nguni ecotypes in southern Mozambique. Trop Anim Health Prod 44:435–444. 10.1007/S11250-011-9915-0 21773680

[CR48] Maciel SMA, Amimo J, Martins M, Mwai AO, Scholtz MM, Neser FWC (2012) Factors influencing reproductive performance of cows from different Nguni ecotypes in southern Mozambique. Trop Anim Health Prod 44:435–444. 10.1007/S11250-011-9915-0 21773680

[CR15] Maciel SMA, Amimo JO, Martins M, Okeyo Mwai A, Scholtz MM, Neser FWC (2013) Feedlot performance of the Nguni in southern Mozambique 10.1007/s11250-011-9915-0 21773680

[CR8] Maciel S, Okeyo AM, Amimo J, Scholtz MM, Neser FWC, Martins M (2013) The effect of geographical region of birth on the reproductive performance of the Nguni in southern Mozambique. S Afr J Anim Sci 43:S60–S63. 10.4314/SAJAS.V43I5.11

[CR47] Macuvele CF, Serafim Vidane A, Birgel DB, Oliveira AC, Nishiyama C, Harry E et al (2025) Reference values and effect of age on hemogram in landim cattle raised in extensive system in districts of Xai-Xai, Limpopo, and Chongoene, Gaza Province, Mozambique. Veterinary Sciences 412:1124. 10.3390/VETSCI12121124 PMC12737348 41472104

[CR20] MADER (2020) Estratégia Agrária de Moçambique 2030 | PDF | Agricultura | Moçambique 2020 https://www.scribd.com/document/662368330/Plano-Estrategico-Para-o-Desenvolvimento-Do-Sector-SUMARIZADA. (Accessed October 6, 2025)

[CR41] Madilindi MA, Banga CB, Bhebhe E, Sanarana YP, Nxumalo KS, Taela MG et al (2020) Genetic differ popul struct four Mozamb indigenous cattle populations 31:1–11

[CR49] Mccarthy N, Cavatassi R, Maggio G (2023) The impact of climate change on livestock production in Mozambique 88

[CR26] Montazeri A, Mohammadi S, M.Hesari P, Ghaemi M, Riazi H, Sheikhi-Mobarakeh Z (2023) Preliminary guideline for reporting bibliometric reviews of the biomedical literature (BIBLIO): a minimum requirements. Syst Rev 12:1–10. 10.1186/S13643-023-02410-2/TABLES/3 PMC10722750 38102710

[CR31] Moreno SG, Sutton AJ, Thompson JR, Ades AE, Abrams KR, Cooper NJ (2012) A generalized weighting regression-derived meta-analysis estimator robust to small-study effects and heterogeneity. Stat Med 31:1407–1417. 10.1002/SIM.4488 22351645

[CR55] Mwai O, Hanotte O, Kwon YJ, Cho S (2015) African Indigenous Cattle: Unique Genetic Resources in a Rapidly Changing World. Asian-Australas J Anim Sci 28:911. 10.5713/AJAS.15.0002R PMC4478499 26104394

[CR6] Ngxumeshe AM, Ratsaka M, Mtileni B, Nephawe K (2020) Sustainable application of livestock water footprints in different beef production systems of South Africa. Sustainability 12:9921. 10.3390/SU12239921

[CR7] Oloo RD, Ojango JMK, Ekine-Dzivenu CC, Gebreyohanes G, Mrode R, Mwai OA et al (2023) Enhancing individual animal resilience to environmental disturbances to address low productivity in dairy cattle performing in sub-Saharan Africa. Front Anim Sci 4:1254877. 10.3389/FANIM.2023.1254877/FULL

[CR45] Otte J, Chilonda P (2003) Classification of cattle and small ruminant production systems in Sub-Saharan Africa. Outlook Agric 32:183–190. 10.5367/000000003101294451;PAGE:STRING:ARTICLE/CHAPTER

[CR43] Paula Pimentel FVDWNJPMAJ (2025) Supplementary feeding increases live weight gain of Angoni cattle during the dry season in Mozambique 2011. https://lrrd.cipav.org.co/lrrd23/6/pime23124.htm. (Accessed December 13, 2025)

[CR21] Pisano M (2020) Planos Nacionais de Adaptação: Lições de Moçambique

[CR33] R Core Team (2025) A language and environment for statistical computing. R Foundation for Statistical Computing, Vienna. - References - Scientific Research Publishin 2022. https://www.scirp.org/reference/referencespapers?referenceid=3456808

[CR1] Rafael José Airone (2012) Effects of body condition, body weight and calf removal on productive and reproductive characteristics of extensive beef cattle in Mozambique

[CR11] Rocha A, Starkey P, Dionisio AC (1991) Cattle production and utilisation in smallholder farming systems in Southern Mozambique. Agric Syst 37:55–75. 10.1016/0308-521X(91)90047-E

[CR46] Rocha A, Starkey P, Dionisio AC (1991) Cattle production and utilisation in smallholder farming systems in Southern Mozambique. Agric Syst 37:55–75. 10.1016/0308-521X(91)90047-E

[CR52] Rocha A, Starkey P, Dionisio AC (1991) Cattle production and utilisation in smallholder farming systems in Southern Mozambique. Agric Syst 37:55–75. 10.1016/0308-521X(91)90047-E

[CR12] Sitoe A (2013) O papel dos recursos naturais renováveis no desenvolvimento sustentável em Moçambique. vol. i

[CR29] St-Pierre NR (2007) Meta-analyses of experimental data in the animal sciences. Revista Brasileira de Zootecnia 36:343–358. 10.1590/S1516-35982007001000031

[CR30] Stanley TD, Doucouliagos H (2015) Neither fixed nor random: weighted least squares meta-analysis. Stat Med 34:2116–2127. 10.1002/SIM.6481 25809462

[CR24] Vaz MAB, Pacheco PS, Seidel EJ, Ansuj AP (2017) Classification of the coefficient of variation to variables in beef cattle experiments. Ciência Rural 47:e20160946. 10.1590/0103-8478CR20160946

[CR3] Vernooij A, Mierlo J, dos Van M (2016) Livestock Development in the Zambezi Valley. Poultry, Dairy and Beef Production, Mozambique

[CR19] Wang X, Xu L (2025) What Are Journals and Reviewers Concerned About in Data Papers? Evidence From Journal Guidelines and Review Reports. Learn Publish 38:e2001. 10.1002/LEAP.2001

[CR9] Woldeyohannes T, Betsha S, Melesse A (2024) Genetic improvement approaches of indigenous cattle breeds for adaptation, conservation and sustainable utilization to changing climate in Ethiopia. Veterinary Integr Sci 22:231–250. 10.12982/VIS.2024.018

[CR51] Wordcloud (2025) Gerador de nuvem de palavras e criador de nuvem de tags online grátis - WordClouds.com 2025. https://www.wordclouds.com/?yygpKSi20tfP1MvMTS8t0kvOz9U3z85ON.K00CvISwcA. (Accessed December 14, 2025)

